# A simple and rapid method for toxicity evaluation of zinc oxide nanoparticle (ZnO NPs) in benthic animal *Hydra magnipapillata*

**DOI:** 10.1016/j.mex.2019.01.003

**Published:** 2019-01-17

**Authors:** Ade Yamindago, Nayun Lee, Seungshic Yum, Seonock Woo

**Affiliations:** a Ecological Risk Research Division, Korea Institute of Ocean Science and Technology (KIOST), Geoje, 53201, Republic of Korea; b Marine Environmental Science, University of Science and Technology (UST), Geoje, 53201, Republic of Korea; c Faculty of Fisheries and Marine Science, Brawijaya University, Malang, 65145, Indonesia; d Marine Biotechnology Research Center, Korea Institute of Ocean Science and Technology (KIOST), Busan, 49111, Republic of Korea

**Keywords:** A method for toxicity evaluation of ZnO NPs in *H. magnipapillata*, Cnidaria, Freshwater animal, Acute toxicity, Morphological changes, Abnormal regeneration

## Abstract

Toxicity evaluation is necessary to investigate the possible risk of chemical or pollutants newly produced such as nanoparticles in the environments. The assessment should apply a method that is effective to determine the toxic concentration and the exposure time of the pollutants in an animal model. This study described three main stages including determining the median lethal concentrations (LC_50_) with Probit program and detecting toxic effects of ZnO NPs in morphology and regeneration observed by the changes in morphology of *Hydra magnipapillata (H. magnipapillata).* We also provide a strategy for culturing hydra in laboratory condition to use the animal for the experiment. The exposure to ZnO NPs led to the abnormality in regeneration such as formation of extraordinary number of tentacles and bifurcated tips in tentacles and the toxic effects in morphology appeared the clubbing tentacle, slender body, and retracting body column and tentacles by the exposure time. The method described here is simple and useful to evaluate the toxic effects of ZnO NPs using morphological characters in *H. magnipapillata* and could suggest the concentration and the exposure time for further investigations on cellular and molecular responses of the animal after exposure to other nanoparticles.

• A simple method to evaluate the toxic effects of ZnO NPs using morphological characters of *H. magnipapillata* and other hydra species.
• A rapid method to evaluate the toxic effects of ZnO NPs and other nanoparticles in *H. magnipapillata*.

## Graphical abstract

**Figure:**
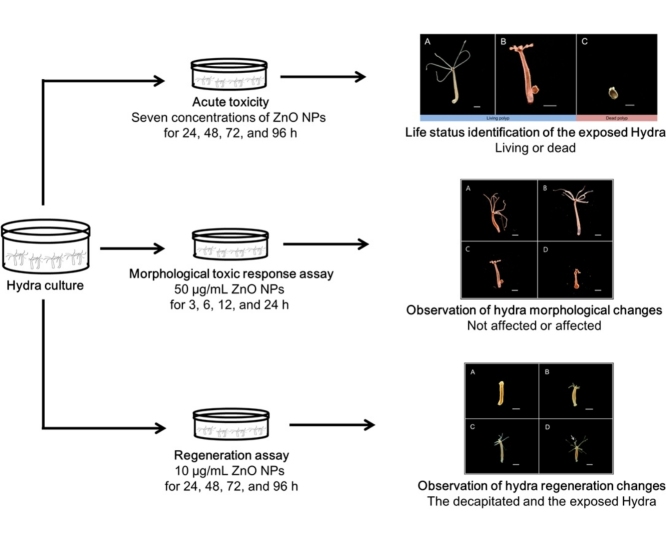

**Specifications Table**

**Table:** 

**Subject Area**	Environmental Science
**More specific subject area:**	Toxicology
**Method name:**	A method for toxicity evaluation of ZnO NPs in *H. magnipapillata.*
**Name and reference of original method**	[1] A. Yamindago, N. Lee, S. Woo, H. Choi, J.Y. Mun, S.-W. Jang, S.I. Yang, F. Anton-Erxleben, T.C.G. Bosch, S. Yum, Acute toxic effects of zinc oxide nanoparticles on *Hydra magnipapillata*, (2018). doi:10.1016/j.aquatox.2018.10.008. [2] S. Yum, S. Woo, A. Lee, H. Won, J. Kim, Hydra, a candidate for an alternative model in environmental genomics, Mol. Cell. Toxicol. (2014). doi:10.1007/s13273-014-0038-3. [3] T. Sugiyama, T. Fujisawa, Genetic analysis of developmental mechanisms in hydra I. Sexual reproduction of *Hydra magnipapillata* and isolation of mutants., Dev. Growth Differ. 19 (1977) 187–200. doi:10:1111/j.1440-169X.1977.00187.x.
**Resource availability**	**Materials**

## Method details

The method presented here provides a simple procedure to determine the lethal concentration and rapid procedures to detect toxic effects of ZnO NPs in morphology and regeneration of *H. magnipapillata* using morphological characters. This method is used to evaluate the acute toxic impacts of ZnO NPs on *H. magnipapillata* [1]. This animal is commonly used as an animal model for biological studies and environmental toxicity tests [2]. It can be cultured in controlled laboratory condition with a simple method previously described [3]. The responses of hydra after exposure to ZnO NPs are specific compared to other nanoparticles (e.g., silicon oxide nanoparticles) [4]. This method suggests the reasonable concentration and the exposure time for investigating the toxic effects of ZnO NPs and other nanoparticles in *H. magnipapillata*. This method will provide important clues for further investigations on the cellular and molecular responses of the animal after exposure to nanoparticles.

## Animal culture procedure

*H. magnipapillata* wild-type strain 105 polyps are cultured as previously described [3]:

1 Prepare hydra culture solution (1 mM NaCl, 1 mM CaCl_2_, 0.1 mM KCl, 0.1 mM MgSO_4_, and 1 mM Tris[hydroxymethyl]aminoethane [pH 7.6]) in a water jar (20 L).
2 Place hydra polyps in cylindrical culture dish (1000 mL) with 500 mL hydra culture solution and incubate in a culture room with an air temperature maintained at 18 ± 1 °C under a photoperiod condition (12 h dark: 12 h light).
3 Before feeding hydra, rinse the freshly hatched *Artemia* nauplii with hydra culture solution twice. The freshly hatched *Artemia* nauplii may be prepared as described in the additional information.
4 Change the culture solution every day, six hours after feeding.

## Acute toxicity assay procedure

**Fig. 1:**
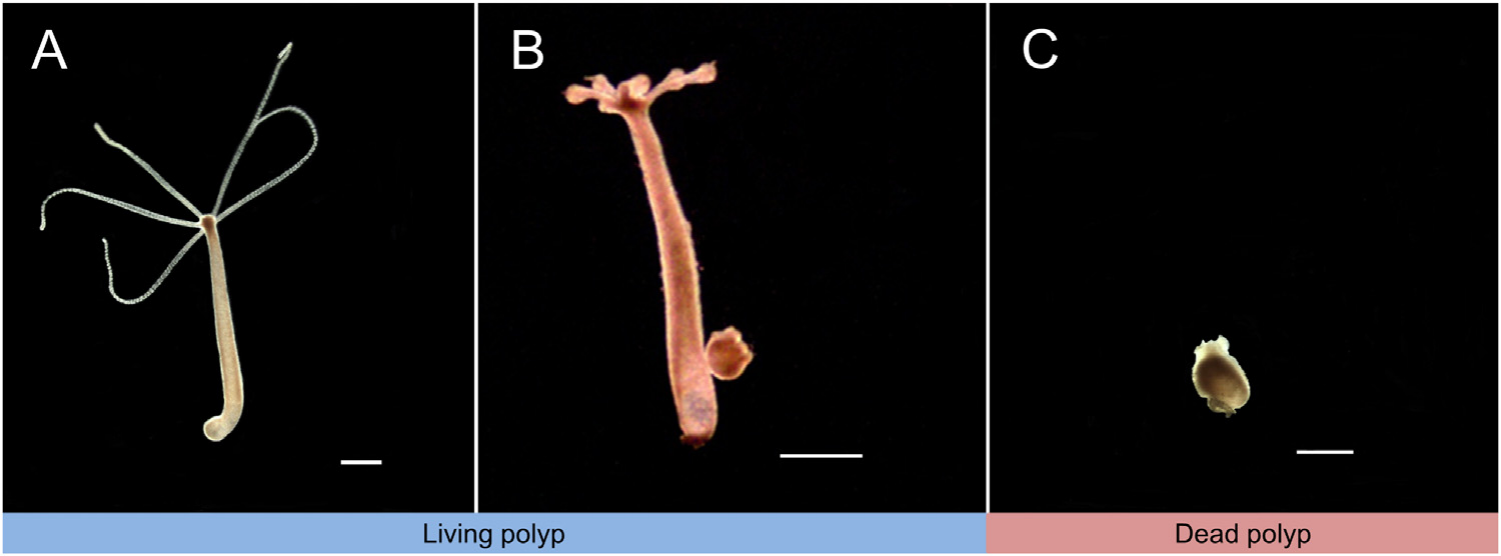
Morphological characters used to identify the lethal condition in *H. magnipapillata* after ZnO NPs exposure. A, Unexposed polyp (living, normal polyp); B, Exposed polyp (living, but tentacles clubbed and body column becoming contracted,); C, Exposed polyp (dead, tentacles and body column becoming totally contracted). Bars, 1 mm.
4 Input the numbers of lethal polyps into a Probit analysis program to determine the LC_50_ concentrations (Table 1). The input process may be performed as described in the additional information.

1 Culture hydra with the culture solution and starve for 48 h before transfer to cell culture dishes.
2 Place 10 polyps in each cell culture dish and add 10 ml hydra culture solution mixed with seven concentrations (10, 25, 50, 100, 150, 200, and 300 μg/mL) of spherical shape ZnO NPs (Ø 20 nm), and prepare a group consisting of 10 polyps in the culture solution without any ZnO NPs as a control. The concentrations were selected after performing preliminary toxicity tests. Incubate the polyps for 24, 48, 72, or 96 h in the culture room. Do not feed the polyps during the toxicity tests.
3 Justify and count the number of lethal polyps based on the morphological characteristics (Fig. 1), and repeat the experiment three times.

**Table 1:** Median lethal concentration of ZnO NPs in *H. magnipapillata*.

Exposure time (h)	LC_50_ (μg/mL, 95% confidence limits)
48	55.3 (27.85–89.96)
72	8.7 (6.79–10.68)
96	7.0 (3.45–8.82)

## Morphological toxic response assay procedure

**Fig. 2:**
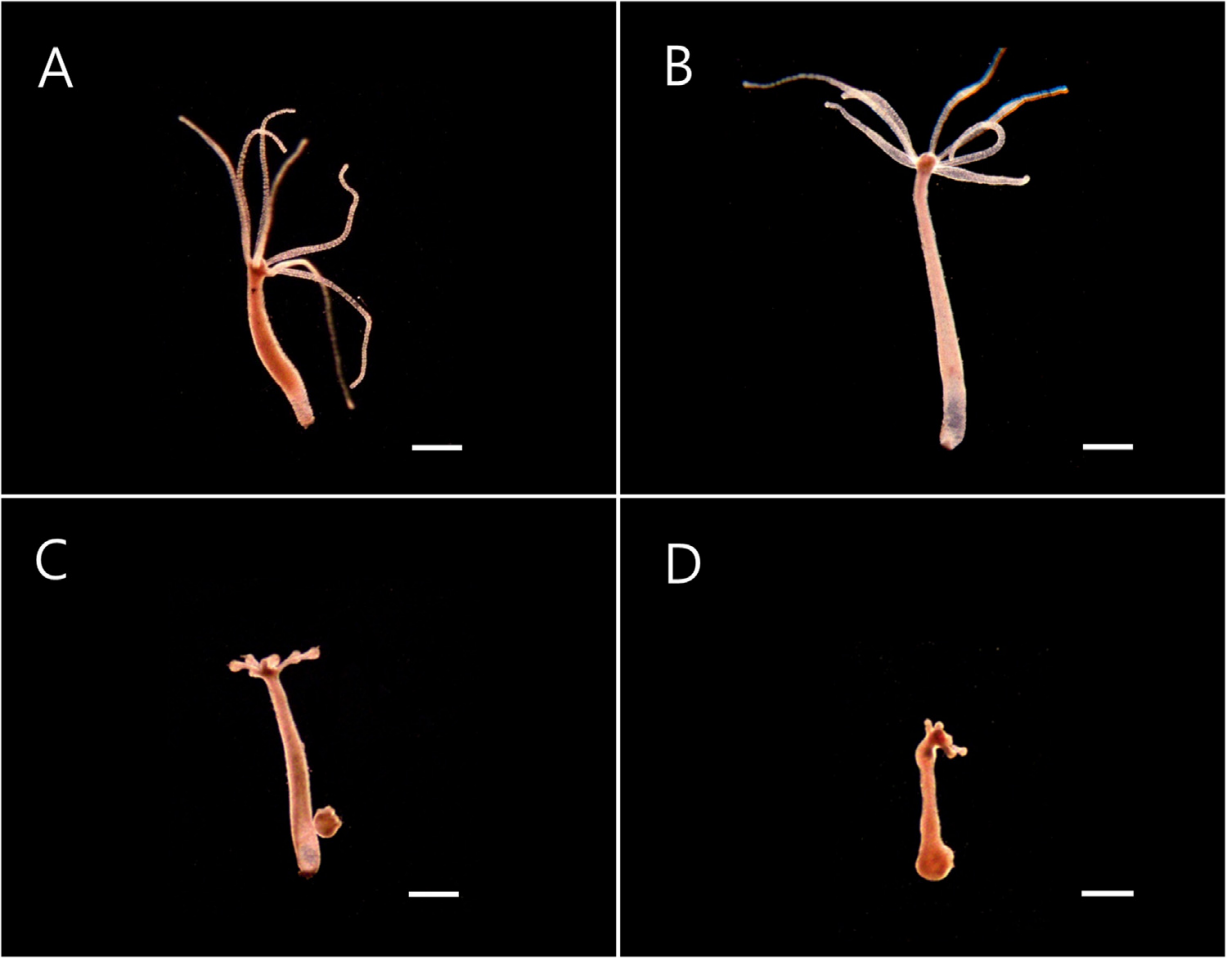
Effects of ZnO NPs in the morphology of *H. magnipapillata* polyps after the exposure to ZnO NPs (50 μg/mL). A, 3 h; B, 6 h; C, 12 h; D, 24 h. Bars, 1 mm.

1 Culture hydra with the culture solution and starve for 48 h before transfer to cell culture dishes.
2 Place ten polyps in each cell culture dish and add 10 ml hydra culture solution mixed with 50 μg/mL ZnO NPs (considering 48 h-LC_50_ of ZnO NPs), and place a group consisting of ten polyps in the culture solution without any ZnO NPs as a control.
3 Incubate all polyps in the culture room, and record the morphological changes after 3, 6, 12, or 24 h using a dissecting microscope (Fig. 2, Table 2). Do not feed the polyps during the toxicity tests.

**Table 2:** Morphological characters for identifying the effects of ZnO NPs in the morphology of *H. magnipapillata*.

Exposure time (h)	Description	Category
3	No morphological changes	Not affected
6	Tentacles starting to club at the tips and body becoming slender	Affected
12	Tentacles are clubbed at the tips	
24	Body column and tentacles are significantly retracted
4 Repeat the experiment three times.

## Regeneration assay procedure

**Fig. 3:**
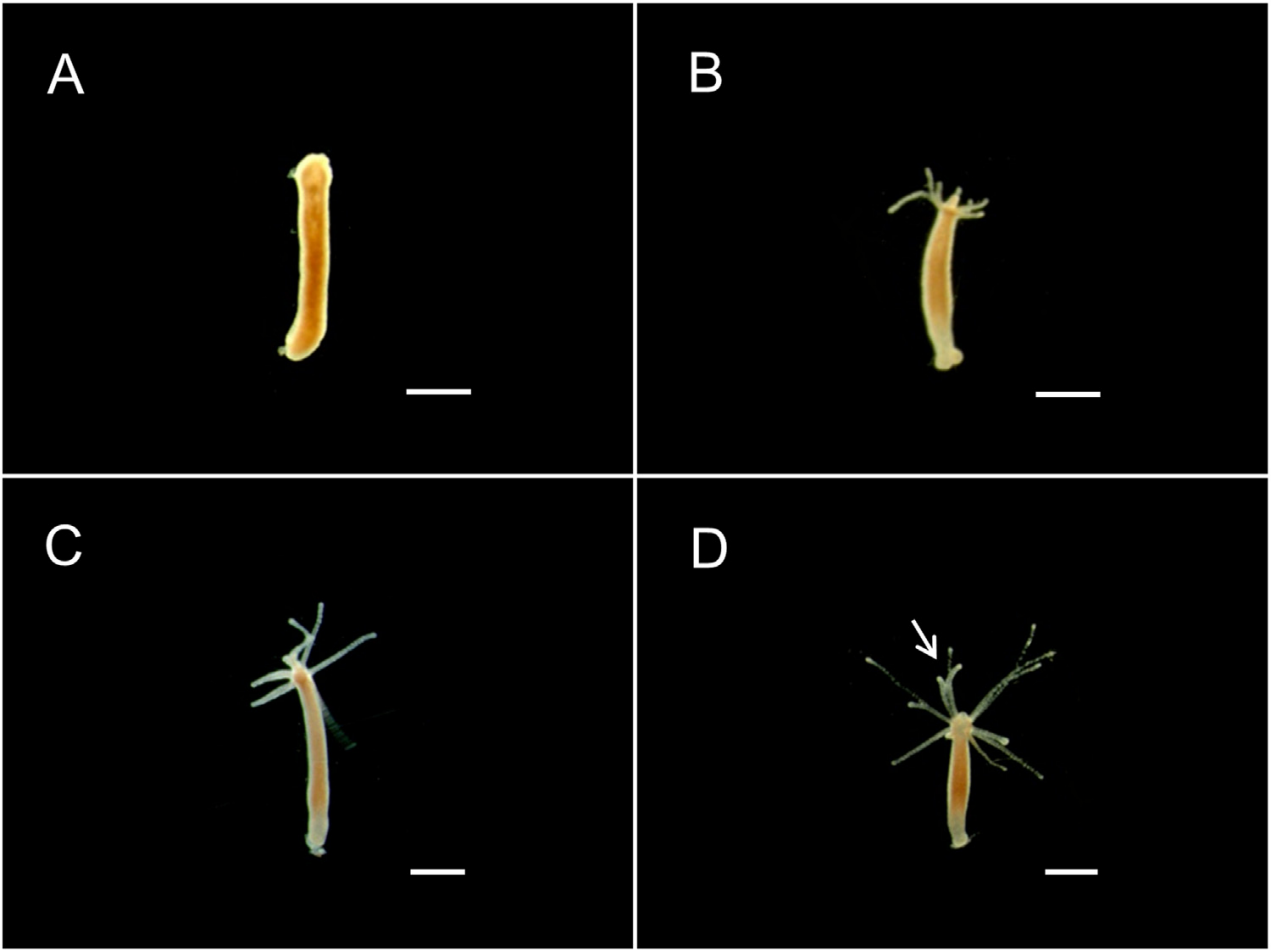
Effects of ZnO NPs in the regeneration of *H. magnipapillata* polyps (10 μg/mL). A, 24 h; B, 48 h; C, 72 h; D, 96 h. Arrow, bifurcated tips. Bars, 1 mm.

1 Culture hydra with the culture solution and starve for 48 h before transfer to cell culture dishes.
2 Place ten polyps in each cell culture dish and add 10 ml hydra culture solution mixed with 10 μg/mL ZnO NPs, and prepare a group consisting of ten polyps with the decapitated head in the culture solution without any ZnO NPs as a control.
3 Incubate all polyps in the culture room and record the regeneration changes after 24, 48, 72, or 96 h using a dissecting microscope (Fig. 3, Table 3). Do not feed the polyps during the toxicity test. In this step, some polyps may survive, but the others may not. The survived polyps lose their tentacles.

**Table 3:** Morphological characters for identifying the effects of ZnO NPs in the regeneration of *H. magnipapillata*.

Exposure time (h)	Description	Category
24	No tentacles	Affected
48	Tentacles started to grow	
72	Tentacles elongated	
96	Abnormal number of tentacles (i.e., 10–11), some tentacles bifurcated at the tips
4 Transfer the survived polyps from the ZnO NPs culture cell into culture cell with 10 mL the culture solution without any ZnO NPs, and let them regenerate.
5 Repeat the experiment three times.

## Additional information

### *Artemia* culture procedure

1 To obtain freshly hatched *Artemia* nauplii, place *Artemia* eggs with 1800 mL natural seawater in a water jar (2000 mL) with aeration and temperature at 25 ± 1 °C.
2 Harvest *Artemia* nauplii after 48 h, and rinse twice with hydra culture solution before feeding hydra polyps.

### Determination of LC_50_ with probit program

1 Prepare the data of dead polyps and run the program.
2 Type “F” to select full input/output and “D” to save the result of analysis as a file. Type file name for output and title of the experiment.
3 Input the number of responding polyps in the control group and number of exposure concentrations. Input the exposure concentrations, the number of responding polyps, and the number of exposed polyps, and verify the data
4 Type “N” to disagree or “Y” to agree with data modification.
5 To view the LC_50_ concentration, find the result of analysis and open it with a word processor program or notepad program.

## Note

The procedures presented here accompany the results described in the previous study about acute toxic effects of ZnO NPs on *H. magnipapillata* [1]. Table 1 was adapted from this study.
